# Maternal Fatigue after Postpartum Anemia Treatment with Intravenous Ferric Carboxymaltose vs. Intravenous Ferric Derisomaltose vs. Oral Ferrous Sulphate: A Randomized Controlled Trial

**DOI:** 10.3390/jcm13030758

**Published:** 2024-01-28

**Authors:** Lea Bombač Tavčar, Hana Hrobat, Lea Gornik, Irena Preložnik Zupan, Marijana Vidmar Šimic, Polona Pečlin, Gorazd Kavšek, Miha Lučovnik

**Affiliations:** 1 Department of Perinatology, Division of Gynecology and Obstetrics, University Medical Centre Ljubljana, Šlajmerjeva 3, 1000 Ljubljana, Slovenia; marijana.vidmarsimic@kclj.si (M.V.Š.); polona.peclin@kclj.si (P.P.); gorazd.kavsek@kclj.si (G.K.); mihalucovnik@yahoo.com (M.L.); 2 Faculty of Medicine, University of Ljubljana, Vrazov trg 2, 1000 Ljubljana, Slovenia; hana.hrobat@icloud.com (H.H.); lea.gornik@gmail.com (L.G.); irena.zupan@kclj.si (I.P.Z.); 3 Department of Hematology, University Medical Centre Ljubljana, Zaloška 7, 1000 Ljubljana, Slovenia

**Keywords:** postpartum anemia, fatigue, intravenous iron treatment, oral iron replacement, patient-centered outcome

## Abstract

(1) **Background:** Postpartum anemia is a common maternal complication and is recognized as a cause of impaired quality of life, reduced cognitive abilities, and fatigue. Efficient iron supplementation for the treatment of postpartum anemia is an essential component of high-quality maternal care. The optimal mode of iron supplementation has not been determined yet, whether oral or intravenous. The objective of this study was to compare postpartum anemia treatment with intravenous ferric carboxymaltose, intravenous ferric derisomaltose, and oral ferrous sulfate. (2) **Methods:** A single-center, open-label, randomized controlled trial. Women with hemoglobin < 100 g/L within 48 h postpartum were randomly allocated to receive intravenous ferric carboxymaltose, intravenous ferric derisomaltose, or oral ferrous sulfate. Intravenous iron was given in one or two doses, while ferrous sulfate was given as two 80 mg tablets once daily. The primary outcome was maternal fatigue measured by the Multidimensional Fatigue Inventory (MFI) six weeks postpartum. Hemoglobin, ferritin, and transferrin saturation levels were analyzed as secondary outcomes. A Kruskal–Wallis test was used for group comparison (*p* < 0.05 significant). (3) **Results:** Three hundred women were included. The MFI score at six weeks postpartum did not differ between groups (median 38 (inter-quartile range (IQR) 29–47) in the ferric carboxymaltose group, median 34 (IQR 26–42) in the ferric derisomaltose group, and median 36 (IQR 25–47) in the ferrous sulfate group; *p* = 0.26). Participants receiving oral iron had lower levels of hemoglobin (135 (131–139) vs. 134 (129–139) vs. 131 (125–137) g/L; *p* = 0.008), ferritin (273 (198–377) vs. 187 (155–246) vs. 24 (17–37) µg/L; *p* < 0.001) and transferrin saturation (34 (28–38) vs. 30 (23–37) vs. 24 (17–37) %; *p* < 0.001) than those receiving ferric carboxymaltose or ferric derisomaltose. (4) **Conclusions:** Intravenous ferric carboxymaltose, intravenous ferric derisomaltose, and oral ferrous sulfate had similar impacts on maternal fatigue at six weeks postpartum despite improved laboratory parameters in the intravenous groups.

## 1. Introduction

Postpartum anemia is a common maternal complication, with a prevalence of up to 50% in developed countries [1,2]. The World Health Organization had defined postpartum anemia as a hemoglobin (Hb) concentration of <100 g/L until it changed its definition to <110 g/L at one week after birth and <120 g/L in the first postpartum year [3]. Anemia in the postpartum period is associated with several adverse health consequences, such as impaired physical work capacity, deficits in cognitive function and mood [4,5,6,7,8,9], and reduced duration of breastfeeding [10]. There are numerous potential causes of anemia in women after childbirth, including blood loss, micronutrient deficiencies such as vitamin A, folate, vitamin B12 and riboflavin deficiency, inherited Hb disorders (e.g., sickle-cell disease and thalassemia), and acute or chronic infections that cause inflammation [3]. Iron deficiency often occurs before anemia and is considered to be the principal cause of anemia in pregnancy and postpartum [1,2,3]. As a result, efficient iron supplementation for the treatment of postpartum anemia is an essential component of high-quality maternal care.

Oral iron supplementation with ferrous sulfate is often recommended as first-line therapy, with blood transfusion reserved for severe cases [11]. However, intravenous iron formulations may be preferred because of low effectiveness, delayed effects, and absorption challenges causing side effects associated with oral iron [12]. In recent decades, several intravenous iron compounds have been registered for clinical use, among which are ferric carboxymaltose and ferric derisomaltose. These formulations were designed to be administered in large doses by rapid intravenous injection. They have been demonstrated to be more efficacious than other iron treatments in patients with inflammatory bowel disease and in patients with chronic kidney disease receiving dialysis [12,13,14,15,16]. In the postpartum period, both ferric carboxymaltose and ferric derisomaltose resulted in a faster rise in Hb levels and other laboratory markers of iron deficiency [6,17,18]. Despite numerous studies evaluating various intravenous and oral iron formulations during pregnancy and postpartum [18], no randomized trial to date has directly compared intravenous ferric carboxymaltose, intravenous ferric derisomaltose, and oral iron for the treatment of postpartum anemia. Most studies comparing different iron formulations published to date have focused mostly on laboratory hematological parameters [18]. It remains to be seen whether ensuring the appropriate treatment of anemia after delivery will result in improvements in the well-being of obstetric patients. Data on the effects of various iron treatments on actual clinical outcomes (such as maternal fatigue) are lacking. Moreover, our manuscript presents the first head-to-head comparison of two novel intravenous iron formulations: ferric carboxymaltose and ferric derisomaltose.

The primary objective of the study was to compare the effects of intravenous ferric carboxymaltose, intravenous ferric derisomaltose, and oral ferrous sulfate treatment of postpartum anemia on maternal fatigue six weeks after childbirth.

## 2. Materials and Methods

### 2.1. Study Design and Participants

A single-center, open-label, active-control, non-inferiority randomized trial was conducted at the Department of Perinatology, University Medical Center Ljubljana, Slovenia. Women with Hb levels between 70 and 100 g/L within 48 h after childbirth were offered to participate in the study. Exclusion criteria were a history of anemia due to known causes other than iron deficiency or blood loss after delivery, active infection defined as clinical signs of infection in combination with increased laboratory inflammatory parameters, hypersensitivity to study medications, renal or hepatic impairment, untreated thyroid deficiency, and pre-existing depression. Women who did not speak Slovene well enough to answer the questionnaire on fatigue were also not included. We did not exclude women with thyroid disorders who were adequately followed and treated by a thyroidologist in the postpartum period.

### 2.2. Randomization and Blinding

Participants were randomized in a 1:1:1 ratio into three groups. Randomization was performed with sequentially numbered (according to a computer-generated random allocation sequence), opaque, sealed envelopes. Intravenous medications were prepared and administered by nurses not involved in the study. 

Participants received intravenous ferric carboxymaltose (Iroprem^®^, Sandoz, Slovenia), intravenous ferric derisomaltose (Monofer^®^, Ewopharma, Schaffhausen, Switzerland), or oral ferrous sulfate (Tardyferon^®^, Pierre Fabre Médicament, Castre, France). For women assigned to the intravenous groups, we calculated the total iron dose needed to correct anemia and replenish iron stores using the Ganzoni formula [19] modified to include adjustment for baseline iron status: Pre-pregnancy weight in kilograms × (15 − baseline Hb) × 2.4 + 500. The calculated amount of intravenous iron administered was rounded to 500 mg (1000, 1500, or 2000 mg). The maximal dose administered in a single day was 1000 mg for ferric carboxymaltose and 1500 mg for ferric derisomaltose as limited by the respective manufacturers. If the total calculated dose exceeded these maximal doses, a subsequent dose was administered in a week. Both intravenous medications were diluted in 250 mL of saline over approximately 15 to 30 min as recommended by the manufacturer. Women assigned to the oral iron group were instructed to take 160 mg of ferrous sulfate once daily (two 80 mg tablets) with fruit juice or vitamin C one hour before meals from inclusion until six weeks postpartum. 

### 2.3. Study Outcomes

The pre-specified primary outcome was a fatigue score extracted from patient-reported fatigue symptoms measured six weeks postpartum by the Multidimensional Fatigue Inventory (MFI) questionnaire. The MFI is a self-report measure requiring between 5 and 10 min for completion. It assesses five dimensions of fatigue: general fatigue, physical fatigue, decreased activity, decreased motivation, and mental fatigue. It consists of 20 statements, for which the participant assesses, on a five-point scale, the extent to which she has been subject to the particular aspect of fatigue in recent days. Higher scores indicate higher levels of fatigue. The obtainable score within each subscale ranges from 4 (absence of fatigue) to 20 (maximum fatigue). In an initial psychometric evaluation, developers reported an internal consistency ranging from 0.53 to 0.93 [20]. It has already been validated in numerous populations, including cancer patients (mean age of 61 years), army recruits (mean age of 21 years), psychology students (mean age of 24 years), and individuals participating in a study of chronic fatigue syndrome (mean age of 39 years) [20,21]. The MFI has also been shown to be a good measure of health-related quality of life after childbirth [22]. 

Secondary study outcomes included:

- MFI physical fatigue score (fatigue score assessed by the physical fatigue domain of the MFI questionnaire) six weeks postpartum;
- Proportion of participants with a high MFI physical fatigue score (physical fatigue score > 15) six weeks postpartum;
- Hb concentration six weeks postpartum;
- Change in Hb concentration from study inclusion to six weeks postpartum;
- Ferritin concentration six weeks postpartum;
- Change in ferritin concentration from study inclusion to six weeks postpartum;
- Transferrin saturation six weeks postpartum;
- Change in transferrin concentration from study inclusion to six weeks postpartum.

Participants were not instructed to fast before the initial or follow-up blood collection. We also assessed the adverse effects of study medications and compliance with the prescribed oral treatment.

### 2.4. Statistical Analysis

The planned sample size of 100 women per group was based on an 80% power to detect non-inferiority using a margin of 5 (half the estimated clinically relevant difference in MFI score), an α of 0.01, and an expected MFI score standard deviation of 10, with a 20% dropout rate.

All data were analyzed according to a pre-established statistical plan in an intention-to-treat fashion. Women who received transfusion due to severe symptoms of anemia after study inclusion and women lost to follow-up six weeks postpartum were not included in the final analysis. Comparisons of continuous variables were performed using a Kruskal–Wallis test and Tamhane’s T2 test for post hoc analysis. Categorical variables were compared using the Chi-square test. A *p* < 0.05 was considered statistically significant. SPSS software (version 24.0; IBM Corporation, Armonk, NY, USA) was used for statistical analysis.

## 3. Results

**Figure 1:**
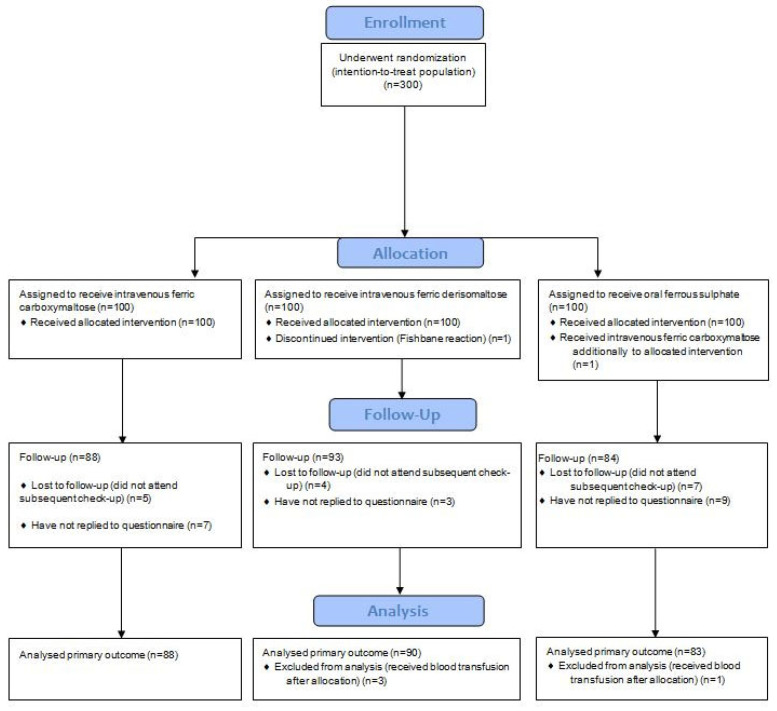
CONSORT diagram for study participation.

A total of 300 women were recruited and randomized between September 2020 and March 2022. Primary outcome data were missing for 35 participants (12%): 16 did not attend the check-up visit at six weeks postpartum and were lost to follow-up and 19 did not fill out the MFI questionnaire at six weeks despite attending the postpartum visit (Figure 1). Four women received red blood cell transfusion shortly after the initiation of iron supplementation due to clinical signs of severe anemia before iron therapy, which could have had an effect. In total, 39 women were excluded from the analysis. All women included were Caucasian. Groups were comparable in terms of demographic parameters and fatigue level at inclusion (Table 1). The median calculated iron deficit at enrolment was similar in both intravenous groups (1422 g in the ferric carboxymaltose group and 1449 g in the ferric derisomaltose group, *p* = 0.19). Baseline MFI total scores, physical fatigue scores, and proportions of participants with severe physical fatigue were similar in the three groups (Table 1). Baseline demographic characteristics and laboratory parameters were similar, with the exception of baseline Hb values that were statistically higher in the ferrous sulfate group (Table 1). Rates of transfusion before inclusion did not differ between groups, with an overall transfusion rate of 6%. 

Table 2 presents a comparison of MFI scores between the study groups. There was no significant difference in total MFI scores at six weeks postpartum among groups. Similarly, there was no significant difference in MFI physical fatigue scores at six weeks postpartum between groups. The proportion of participants suffering from severe physical fatigue (MFI physical score > 15) was also not significantly different among groups. In all treatment groups, total MFI scores, MFI physical fatigue scores, and proportion of severe physical fatigue decreased from baseline at study inclusion to follow-up at six weeks postpartum. However, the rate of decrease did not differ significantly among groups.

A comparison of hematological laboratory parameters between groups is shown in Table 3. Concentrations of Hb, ferritin, and transferrin saturation differed significantly. Hb levels were significantly higher in the intravenous ferric carboxymaltose and intravenous ferric derisomaltose groups compared to the oral ferrous sulfate group. There were no significant differences in Hb concentrations at six weeks postpartum between the two intravenous iron groups (*p* = 0.90). Similarly, ferritin concentrations were significantly higher in the intravenous vs. oral iron groups. Ferritin concentrations were higher in the intravenous ferric carboxymaltose vs. intravenous ferric derisomaltose group (*p* < 0.01). 

Changes in Hb, ferritin, and transferrin saturation from study inclusion to six weeks postpartum differed significantly among groups. Changes in all three parameters were significantly higher in the intravenous ferric carboxymaltose and intravenous ferric derisomaltose groups compared to the oral ferrous sulfate group. Increases in Hb and transferrin saturation did not differ significantly between the intravenous ferric carboxymaltose and intravenous ferric derisomaltose groups (*p* = 0.93 and *p* = 0.18, respectively). Ferritin increase was larger in the intravenous ferric carboxymaltose compared to the intravenous ferric derisomaltose group (*p* < 0.01). 

Forty (48%) women randomized to receive oral ferrous sulfate reported adverse effects related to, or possibly related to, study medication. Most of these (30; 75%) reported gastrointestinal side effects, such as nausea, diarrhea, and obstipation. Ten (12%) women in the ferrous sulfate group discontinued this medication before their six-week postpartum visit due to constipation. 

Urticarial rash or general pruritus without rash was noted in four participants while receiving intravenous ferric carboxymaltose, which resolved spontaneously. In the ferric derisomaltose group, there was one case of skin hyperpigmentation at the site of infusion due to medication extravasation and one case of a Fishbane reaction [23] with lumbar musculature spasm, which resolved spontaneously within minutes of stopping the application. There were no cases of severe anaphylaxis with hemodynamic compromise or angioedema in any of the participants.

## 4. Discussion

We found no significant association between the type of iron medication used to treat postpartum anemia and maternal fatigue level six weeks after childbirth. Intravenous iron substitution with ferric carboxymaltose or ferric derisomaltose resulted in higher body iron stores with higher Hb concentrations at six weeks after childbirth compared to oral ferrous sulfate treatment. 

Our results confirm that effective iron therapy, regardless of the medication type and route of administration, effectively corrects anemia and is associated with a reduction in maternal fatigue postpartum. This is in accordance with the findings of Van der Wyck et al. [24], who showed that early postpartum initiation of iron therapy, either intravenous or oral, improved health-related quality of life in women with postpartum anemia.

Our findings are in contrast with two previous studies comparing clinical outcomes in postpartum women treated with intravenous vs. oral iron. Holm et al. [6] found a single dose of intravenous ferric derisomaltose to be associated with a significant reduction in maternal physical fatigue within 12 weeks after delivery when compared to oral iron supplementation. Similarly, Westad et al. [25] reported lower total fatigue scores at 4, 8, and 12 weeks postpartum in anemic women treated with intravenous ferric sucrose, compared to standard oral iron therapy. 

Consistent with our findings, several studies demonstrated a greater and faster rise in Hb and iron stores with intravenous compared to oral iron treatments [6,18,24,25,26,27,28,29]. Significantly higher Hb concentrations were observed in the intravenous ferric carboxymaltose group as compared to the oral iron groups postpartum in numerous studies [18,24,25,26,27,28,29]. In contrast, a comparison of intravenous ferric derisomaltose and oral iron for the treatment of postpartum anemia is limited to only one study published to date. Holm et al. [6] reported a larger increase from the baseline Hb level in the ferric derisomaltose group compared to oral iron. 

Despite being more effective in correcting hematological values, intravenous iron formulations did not significantly impact the patient-oriented outcome, i.e., postpartum maternal fatigue. The lack of effects of intravenous vs. oral iron treatments on maternal fatigue in our study could be explained by numerous factors that impact the sense of fatigue besides anemia. Sleep deprivation, pain, and postpartum mood disorders have all been associated with maternal fatigue and were not accounted for in the present study [30,31].

The study was not powered to detect differences in secondary outcomes, such as Hb, ferritin, and transferrin levels. The lack of differences in such outcomes between the two intravenous iron groups should not be over-interpreted and needs to be confirmed or refuted by further studies.

This randomized trial was adequately powered to detect potential differences in a clinically relevant outcome, i.e., postpartum maternal fatigue. We consider this a strength of the study since it did not primarily aim to evaluate changes in laboratory values alone, as was the case with several studies published in the field of pregnancy or postpartum anemia treatments so far [18,24,25,26,27,28,29]. Maternal fatigue is an important clinical outcome associated with overall maternal physical and mental well-being and mother–child interaction [8]. Another strength of the study is the relatively narrow time window (48 h after childbirth) for participant enrollment. This ensured the homogeneity of the study population in terms of physiological states, such as systemic inflammation after childbirth. To the best of the authors’ knowledge, this is the first study on the effects of different iron treatments for postpartum anemia with a head-to-head comparison of intravenous ferric carboxymaltose and intravenous ferric derisomaltose. 

Several limitations of the study should also be considered. First, there was no placebo control group. Therefore, it is not possible to attribute decreases in fatigue scores and increases in hematological parameters to iron treatments with certainty. Nevertheless, given the large body of evidence on adverse health consequences of untreated postpartum anemia [1,11], the inclusion of a placebo arm was deemed unethical. Second, the study was not blinded. It is possible that participants had different expectations of different treatments, which may have biased the MFI questionnaire results. Blinding in the present study would not have been possible due to the different routes of administration of study medications. While it would have been technically possible to blind the two parenteral iron formulations, the study would still have to be considered an open-label trial, as not all study treatments could have been blinded. Although investigators were aware of group allocation, this did not influence the results, since outcomes were either participants’ self-reported fatigue or laboratory values. The third limitation of the study is the relatively short observation period (six weeks). As a result, anemia-related fatigue symptoms that would manifest later would not have been detected. Fourth, the study only focused on iron supplementation in postpartum women with anemia. Since fatigue can be caused by iron deficiency independent of anemia, further studies will be needed to assess the effects of iron treatments in women with higher Hb levels after birth. Fifth, the use of a generic questionnaire for the assessment of fatigue may also be considered a limitation. As there are no existing questionnaires developed specifically for postpartum women, we chose to use the most appropriate validated generic questionnaire, which has already been used in studies evaluating postpartum maternal fatigue [6]. Sixth, the diversity of the study population was limited as all women included in the study were Caucasian. Seventh, it is possible that if the alternate-day treatment for oral iron was used in the trial, the results might be different since there are data indicating that oral iron therapy is more effective when given every other day due to the effect of hepcidin on iron absorption [32]. Finally, baseline Hb levels were higher in the oral ferrous sulfate group compared to the two intravenous iron groups. Given the adequate randomization technique, we attribute this to chance. However, the main findings were most probably not influenced by this fact, especially considering similar fatigue reductions from baseline in all three study groups.

## 5. Conclusions

In conclusion, we find that despite improved hematological laboratory parameters in the intravenous iron groups, maternal fatigue at six weeks postpartum was not impacted by the type of iron medication used to treat postpartum anemia.

## Figures and Tables

**Table 1:** Participant demographics and baseline characteristics.

Statistics/Category	Treatment Group			*p* Value
	Intravenous Ferric Carboxymaltose (*n* = 88)	Intravenous Ferric Derisomaltose (*n* = 90)	Oral Ferrous Sulphate (*n* = 83)	
Maternal age, years	32 (28–36)	30 (27–35)	31 (28–36)	0.237
Prepregnancy BMI, kg/m^2^	24 (21–27)	24 (22–27)	23 (21–27)	0.631
BMI at delivery, kg/m^2^	28 (27–32)	30 (27–32)	29 (26–32)	0.485
Multiple gestation	6 (2)	6 (2)	2 (1)	0.351
Nulliparity	58 (22)	64 (25)	46 (18)	0.092
Gestational age at childbirth, completed weeks	39 (38–40)	39 (38–40)	39 (38–40)	0.590
Transfusion of RBC before intervention	5 (2)	5 (2)	7 (3)	0.691
Cesarean section	28 (11)	22 (8)	28 (11)	0.365
Maternal level of education *	8 (5–9)	8 (5–9)	8 (5–9)	0.465
Calculated iron deficit †, g	1422 (1281–1567)	1449 (1346–1586)	/	0.191
Hemoglobin, g/L	92 (87–97)	91 (87–95)	95 (91–98)	0.006
Ferritin, µ/L	29 (11–47)	20 (12–30)	22 (13–41)	0.106
Transferrin saturation, %	9 (7–14)	10 (7–15)	10 (7–16)	0.301
Plasma iron µmol/L	6 (4–8)	8 (5–12)	7 (5–11)	0.163
TIBC, µmol/L	71 (64–77)	73 (67–80)	71 (65–80)	0.334
CRP, mg/L	57 (36–94)	53 (35–78)	48 (34–76)	0.576
Total MFI score	49 (41–62)	48 (34–60)	51 (37–62)	0.340
Physical fatigue MFI score	12 (8–15)	11 (8–15)	12 (8–15)	0.816
Physical fatigue MFI score > 15 ‡	27 (31)	23 (28)	20 (26)	0.713

Data are presented as median (interquartile range) or *n* (%); BMI, body mass index; RBC red blood cells; TIBC, total iron-binding capacity; CRP, C-reactive protein; MFI, Multidimensional Fatigue Inventory; * educational attainment using the Statistical Office of the Republic of Slovenia grading (from 0, no education; 12, PhD degree); † Iron deficit calculated using Ganzoni formula; ‡ physical fatigue MFI score > 15 indicates severe physical fatigue.

**Table 2:** Comparison of Multidimensional Fatigue Inventory (MFI) scores between the three study groups.

Statistics/Category	Treatment Group			*p* Value
	Intravenous Ferric Carboxymaltose (*n* = 88)	Intravenous Ferric Derisomaltose (*n* = 90)	Oral Ferrous Sulphate (*n* = 83)	
Total MFI score six weeks postpartum	38 (29–47)	34 (26–42)	36 (25–47)	0.26
Decrease in total MFI score from study inclusion to six weeks postpartum	12 (4–22)	12 (4–20)	11 (1–21)	0.76
Physical fatigue MFI score six weeks postpartum	8 (5–11)	7 (5–10)	8 (5–11)	0.10
Decrease in physical fatigue MFI score from study inclusion to six weeks postpartum	3 (0–6)	3 (0–6)	3 (0–6)	0.78
Physical fatigue MFI score > 15 * six weeks postpartum	7 (8)	5 (6)	5 (6)	0.79
Decrease in proportion of participants with physical fatigue MFI score > 15 from study inclusion to six weeks postpartum	20 (23)	18 (20)	15 (18)	0.75

Data are presented as median (interquartile range) or n (%); * physical fatigue MFI score > 15 indicates severe physical fatigue.

**Table 3:** Comparison of hematological laboratory parameters between the three study groups.

Statistics/Category	Treatment Group			*p* Value
	Intravenous Ferric Carboxymaltose (*n* = 88)	Intravenous Ferric Derisomaltose (*n* = 90)	Oral Ferrous Sulphate (*n* = 83)	
Hemoglobin at six weeks postpartum, g/L	135 (131–139)	134 (129–139)	131 (125–137)	0.008
Change in hemoglobin from study inclusion to six weeks postpartum, g/L	43 (37–51)	45 (38–50)	36 (31–44)	<0.001
Ferritin at six weeks postpartum, µg/L	273 (198–377)	187 (155–246)	24 (17–37)	<0.001
Change in ferritin from study inclusion to six weeks postpartum, µg/L	240 (173–336)	163 (127–219)	0 (−10–12)	<0.001
Transferrin saturation at six weeks postpartum, %	34 (28–38)	30 (23–37)	24 (17–37)	<0.001
Change in transferrin saturation from study inclusion to six weeks postpartum, %	23 (18–28)	19 (10–28)	12 (5–24)	<0.001

Data are presented as median (interquartile range).

## Data Availability

The datasets used and analyzed during the current study are available from the corresponding author on reasonable request in the format requested. Data will be available on request from publication onward for at least 15 years.
